# Observation of the clinical effect of immediate implantation and delayed implantation in the maxillary and maxillary molars

**DOI:** 10.4314/ahs.v23i2.69

**Published:** 2023-06

**Authors:** Jie Yu, Xiaoping Liu, Daning Li, Yiming Xu, Chao Wang

**Affiliations:** Department of Stomatology, the 80^th^ Group Army Hospital of the Chinese People's Liberation Army, Weifang 261021, Shandong, China

**Keywords:** Aesthetics, alveolar bone resorption, delayed implantation, immediate implantation, molars

## Abstract

**Objective:**

To study the clinical application effect of two kinds of implants in the upper and lower molars.

**Methods:**

A selection of 120 patients (134 teeth) who underwent implant treatment in the upper and lower molars in the army hospital of the Chinese people's liberation army from January 2018 to June 2019 were divided into an immediate group (using immediate implantation) and a delayed group (using delayed implantation) using a random number table 60 cases (60 teeth) in each group; differences in implant success rate, buccal keratinized gingival width before and after treatment, alveolar bone absorption, periodontal pocket depth, and gingival aesthetic indicators were compared between the two groups.

**Results:**

The gingival aesthetics effect of the immediate group was better than that of the delayed group on the whole and the difference was statistically significant (P<0.05); after 12 months of restoration, the implantation success rate of the immediate group was 96.67%. The deferred group was 93.33%, and the difference between the two groups was not statistically significant (P>0.05).

**Conclusion:**

Both delayed restoration and immediate implant restoration can achieve good results in implant restoration treatment in the maxillary and maxillary molars. However, immediate implantation has certain advantages in reducing the amount of alveolar bone absorption and maintaining the aesthetic effect of the gums.

## Introduction

With the increase of age and other events, resulting in a significant increase in the number of people receiving dental implants in the upper and lower molar areas every year. The clinical implants are recommended to restore dental function for such populations 1-2. Implant therapy has two forms, one is delayed planting, and the other is immediate planting. The molar area is the main area responsible for chewing function, and prompt treatment will help improve the chewing function and quality of life of patients 3-4.

Delayed implant was the implementation of implant placement surgery 3 to 6 months after tooth extraction, and it needs to wait 3 to 6 months after tooth extraction to wait for wound healing 5. The treatment cycle of this program is very long. During the waiting period, alveolar bone will be absorbed in large quantities, which can cause gingival atrophy and affect the repair effect 6. Immediate implantation is the treatment of implant placement after tooth extraction, which has the advantages of short treatment cycle and high safety. In recent years, immediate implantation has gradually been widely accepted in clinic 7-8. At present, there are few reports on the effect of immediate implant treatment on reducing alveolar bone absorption and maintaining gingival reducing alveolar bone absorption and maintaining gingival aesthetic effect. In the present investigation the data of immediate implant cases in the molar regions completed in the Department of Dental Implantation of our hospital in recent years, and takes the cases with delayed implant in the molar regions as the control group has been discussed. The analysis is carried out from the aspects of buccal gingival width, alveolar bone absorption, periodontal pocket depth, and gingival aesthetic index, so as to provide reference for the selection and formulation of surgical schemes for clinical implant treatment.

## Materials and methods

### Information

A total of 120 patients (120 teeth) with maxillary and mandibular molar implant treatment in our hospital from January 2018 to June 2019 were selected. The random number table was used to divide them into the immediate group (immediate planting) and the delayed group (delayed planting) with 60 teeth in each group. Inclusion criteria: (1) The first and second molars of the upper and lower jaws cannot be retained due to periodontal disease and root fracture; (2) The age of the research object is 19–55 years old; (3) The completion of molar implantation by the same dentist; (4) being able to receive referral on time; (5) The research programme is approved by the Medical Ethics Committee and signed an informed consent with the patient himself. Exclusion criteria: (1) mental illness; (2) Oral and gingival inflammation, infectious diseases; (3) Serious tissue defects in the proposed planting area; (4) Scar constitution; (5) Tumor patients.

There were 60 patients (66 teeth) in the immediate group, aged from 19 to 51 years, with an average age of 38.2 ± 9.7 years, including 36 males and 24 females. There were 60 patients (68 teeth) in the delayed group, aged from 19 to 55 years old, with an average age of 39.4 ± 10.4 years old, including 31 males and 29 females. There was no significant difference in age and gender between the two groups (P> 0.05).

### Planting repair method

Immediate implant surgery has been done before filming panoramic teeth, cleaning teeth, oral cephalosporin antibiotics, and containing ciclopramide tablets. Under local anesthesia, minimally invasive technique was used to remove the affected teeth, clean the alveolar fossa, repair the bone wall and the alveolar crest of the protrusion, and prepare the implant socket at the middle of the alveolar. When there is a defect around the implant, it is necessary to add bone powder, cover the dental restoration film at the bone graft, and close suture. After surgery, oral cephalosporins and tinidazole for anti-infective treatment, including oral ciclopramide tablets, and 1 week after removal of the line has been done.

The extraction of delayed implant teeth was consistent with the immediate implant group. Three months later, implant surgery. The method of implantation was the same as immediate implantation. After 6 months and 12 months of implantation, follow-up examination and photography were performed to observe the bone remodelling and bone integration around the implant.

### Observation indicators and evaluation methods

The success rate of implant in the two groups was compared (one year after operation, no implant loosening, shedding, no transmission around the implant by X-ray examination, and the vertical bone absorption within 1 year after operation (<2.0 mm). The width of buccal keratinized gingival and the depth of periodontal pocket was measured at the time of permanent restoration, 6 months after restoration and 12 months after restoration. The alveolar bone changes were detected and compared between the two groups, and the mesial bone absorption was calculated after 6 months and 12 months of repair.

The gingival aesthetic effects of the two groups were compared. The gingival texture, gingival color, alveolar crest defect, gingival margin shape and gingival height were observed, no gingival papilla (grade 0), gingival papilla lower than normal 1/2 (grade 1), gingival papilla higher than normal 1/2, adjacent space not filled (grade 2), gingival papilla adjacent space filled, consistent with adjacent dental papilla (grade 3), gingival papilla hyperplasia, covering part of the crown (grade 4) 9.

Buccal keratinized gingival width: The data were measured immediately after implant restoration and during follow-up. Periodontal probe was used to measure the distance from the gingival margin to the gingival union at the center of buccal side in the long axis direction of the implant, and the average value was measured for three times 10.

Bone resorption at the proximal and distal edge of the implant: According to the imaging examination, the distance from the highest point of the distal bone union crown of the implant to the distal shoulder of the implant was measured. Three measurements were repeated, and the average value of the three measurements was taken. And according to the ‘actual marginal bone level = bone level measurement value* actual implant length/ implant length measurement value’ to calculate the bone absorption of the two measurement time points 11.

The parallel projection technique was used to take the dental film. The crown edge of the implant neck and the crown point of the bone-implant contact interface were taken as the reference. The near-mid and far-mid sites were taken. The distance between the two points was measured and the difference was calculated, namely, the vertical bone absorption at the implant edge 12.

The periodontal pocket depth was detected by pure titanium periodontal probe, and the distance from the near-middle, central and far-middle mucosal edges of the labial and lingual crowns to the bottom of the pocket was detected. The average value was measured twice 13.

### Statistical analysis

SPSS 21.0 software was used for data processing. In this study, the buccal keratinized gingival width and periodontal pocket depth were tested by normal distribution, they all conform to the approximate normal distribution or normal distribution, which is represented by (x=±s), and the t test is used for data comparison, χ^2^ test was used for comparison between groups of enumeration data ,Mann-Whitney U test was used for comparison between groups of grade counting data ; Test level α = 0.05.

## Results

### Comparison of general data

The age, gender, BMI, smoking, drinking and planting sites of patients in the immediate and delayed groups were statistically analysed, and the differences between the two groups were not statistically significant (P>0.05) (Table 1).

**Table 1:** General information comparison

Normal information	Immediate group (n=60)	Deferred Group (n=60)	t/χ^2^	P
Age (years)	38.2±9.7	39.4±10.4	-0.654	0.515
BMI (kg/m^2^)	23.9±2.2	24.1±2.4	-0.476	0.635
Gender (%)			0.845	0.358
Male	36(60.00)	31(51.67)		
Female	24(40.00)	29(48.33)		
Smoking (%)			0.833	0.361
Yes	14(23.33)	10(16.67)		
No	46(76.67)	50(83.33)		
Drinking (%)			1.915	0.166
Yes	22(36.67)	15(25.00)		
No	38(63.33)	45(75.00)		
Location (%)			1.212	0.271
Maxillary molars	30(50.00)	36(60.00)		
Mandibular molars	30(50.00)	24(40.00)		

### Comparison of near-middle bone resorption between the two groups after repair treatment

In the permanent restoration, there was no statistically significant difference in the buccal keratinized gingival width and periodontal pocket depth between the immediate group and the delayed group (P>0.05). There was no significant difference in buccal keratinized gingival width between the two groups after 6 months and 12 months of repair (P> 0.05). The depth of periodontal pocket in the immediate group was less than that in the delayed group after 6 months and 12 months of repair, and the difference was statistically significant (P<0.05) (Table 2).

**Table 2:** Comparison of changes in buccal keratinized gingival width and periodontal pocket depth before and after prosthetic treatment between the two groups (x̅±s)

Index	Group	When permanently repairing	6 months after repair	12 months after repair
Buccal keratinized gingival width (mm)	Immediate group (n=60)	4.94±0.75	4.88±0.74	4.82±0.76
	Deferred Group (n=60)	5.02±0.80	4.75±0.81	4.64±0.84
	t	-0.565	0.918	1.231
	P	0.573	0.361	0.221
Periodontal pocket depth (mm)	Immediate group (n=60)	1.38±0.40	1.72±0.48	1.83±0.37
	Deferred Group (n=60)	1.31±0.32	1.96±0.41	2.05±0.35
	t	1.059	-2.945	-3.346
	P	0.292	0.004	0.001

### Comparison of near-middle bone resorption between the two groups after repair treatment

After 6 months and 12 months of repair, the measured value of mesial bone absorption in the immediate group was less than that in the delayed group, and the difference was statistically significant (P<0.05) (Table 3).

**Table 3:** Comparison of mesial bone resorption after repair treatment between the two groups (x̅±s, mm)

Group	n	6 months after repair	12 months after repair
Immediate group	60	0.52±0.14	0.72±0.21
Deferred Group	60	0.60±0.16	0.85±0.25
t		-2.915	-3.084
P		0.004	0.003

### Comparison of gingival aesthetic indexes between the two groups after restoration treatment

The gingival aesthetic index was evaluated 12 months after restoration, and the gingival aesthetic effect of the immediate group was better than that of the delayed group on the whole, and the difference was statistically significant (P<0.05) (Table 4).

**Table 4:** Comparison of aesthetic indexes of gingival between two groups of patients after restorative treatment [n (%) ]

Group	n	0 grade	1 grade	2 grade	3 grade	4 grade
Immediate group	60	0(0.00)	2(3.33)	21(35.00)	34(56.67)	3(5.00)
Deferred Group	60	2(3.33)	8(13.33)	28(46.67)	22(36.67)	0(0.00)
Z		-3.238				
P		0.001				

### Comparison of implant success rate between two groups

After 12 months of repair, the success rate of implantation was 96.67 % in the immediate group and 93.33 % in the delayed group. There was no significant difference between the two groups (P>0.05) (Table 5).

**Table 5:** Comparison of implant success rate between the two groups [n (%)]

Group	n	Planted successfully	Planting failure
Immediate group	60	58(96.67)	2(3.33)
Deferred Group	60	56(93.33)	4(6.67)
χ^2^		0.702	
P		0.402	

### Typical cases

**Figure 1:**
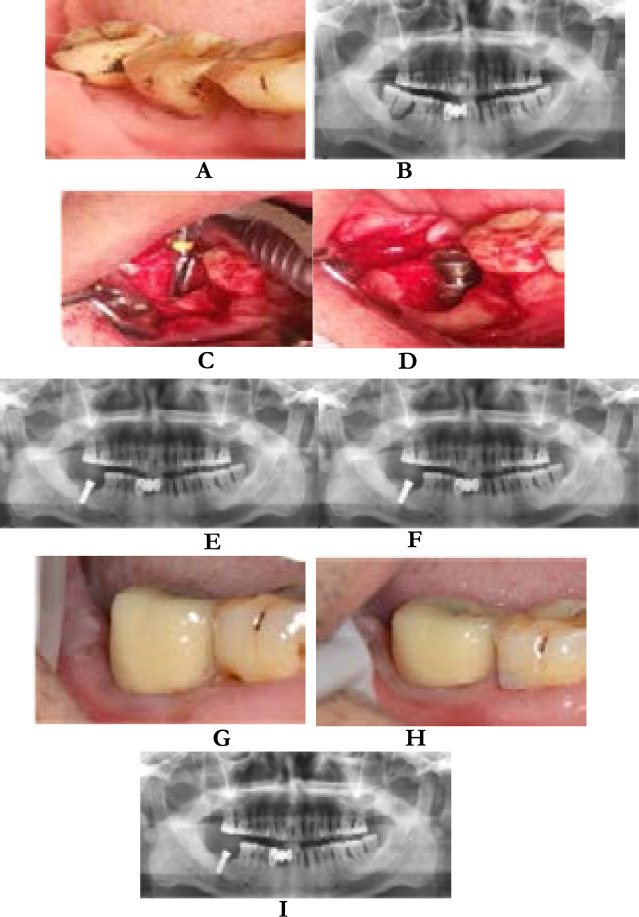
A is a preoperative intraoral photo, B is a preoperative curved tomography, C is a stepwise hole preparation, D is implanting placement, E is an immediate curved tomography of the implant, F is a curved tomography during restoration, and G is a restoration during restoration Intraoral photos, H and I are intraoral photos and curved tomograms at 12-month follow-up, respectively

A 48-year-old male patient presented with right mandibular molar loosening for 2 years. Recently, gingival swelling and pain aggravated, affecting masticatory function. The patient was diagnosed as 47 and 48 periodontal-endodontic lesions by experts. Immediate implantation was performed on the 47^th^ molar, and the 48^th^ molar was removed. After 12 months of repair, the patient did not appear discomfort, gingival swelling and pain, implant stability, masticatory function was good, and some imaging data (Figure 1).

**Figure 2:**
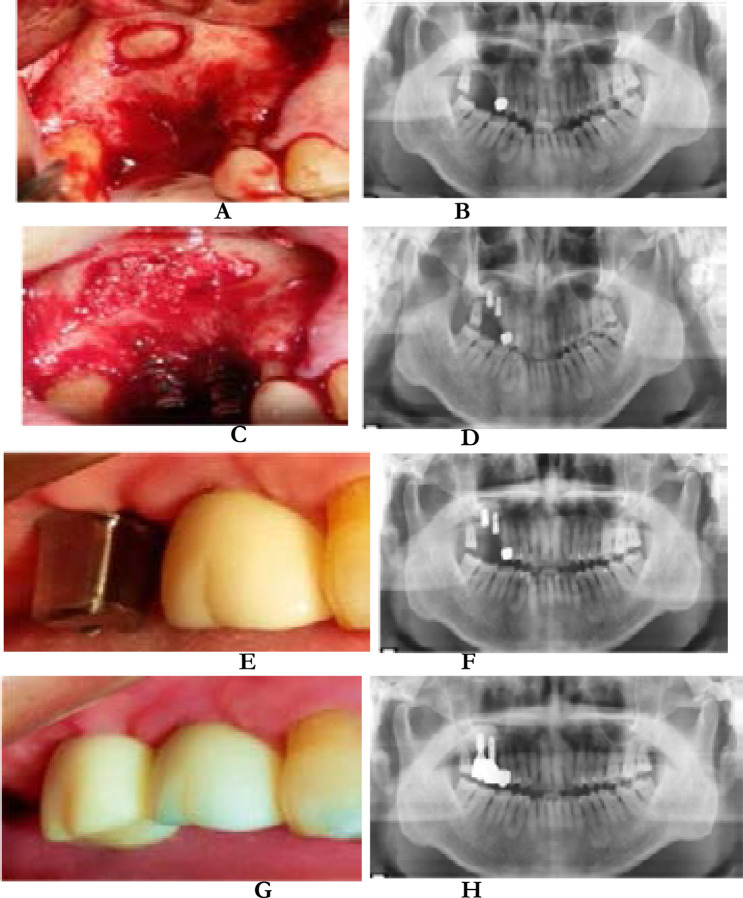
A and B are intraoral and curved tomographic films of the preparation of rectangular bone windows, C and D are intraoral and curved tomographic films when implants are implanted and bone grafted, E and F are intraoral and curved tomographic films before restoration, G And H are the intraoral photos and curved tomograms taken during the 12-month follow-up, respectively

A 54-year-old male patient with right maxillary molars was required to undergo implant restoration after 6 months of extraction. The patient was diagnosed with dentition defect by experts on admission. The delayed implant scheme was adopted. After 12 months of restoration, the patient did not appear discomfort, gingival swelling and pain, implant stability, good masticatory function, and no complications such as food incarceration. The patient was satisfied with some imaging data (Figure 2).

## Discussion

The traditional concept of dental implant restoration believes that the implant should be implanted after the complete healing of the extracted wound, which needs to wait at least three months. Until recent years, people pay attention to immediate implant therapy. Delayed implant waiting period, bone mass and soft tissue mass will be lost, affecting the recovery after implant implantation 14. In addition, immediate implantation reduces the number of operations, shortens wound healing time and improves the stability of soft and hard tissues. Fresh tooth extraction has the effect of guiding the direction of implant, while retaining the bone and keratinized gingival to the greatest extent, which can ensure the long-term efficacy of implant restoration 15.

The buccal keratinized gingival of the implant can resist the physical stimulation caused by food during daily chewing, buffer the traction of muscles and ligaments around the mouth, reduce the mobility of soft tissue, and maintain the integrity and stability of biological closure around the implant. Studies have shown that 16-17 insufficient buccal keratinized gingival width will significantly increase the risk of soft tissue atrophy and connective tissue attachment loss after repair. In severe cases, the edge of the implant will be exposed, which not only reduces the bone level at the edge of the implant, but also affects the appearance. This effect increases the incidence of discomfort during tooth brushing and increases the difficulty of oral hygiene maintenance. In this study, after 6 months and 12 months of restoration, there was no significant difference in the width of buccal keratinized gingival between the two groups, suggesting that immediate planting and delayed planting had similar effects on maintaining the width of buccal keratinized gingival.

After 6 and 12 months of repair, the depth of periodontal pocket in the immediate group was significantly lower than that in the delayed group. The results showed that the immediate implant soft tissue was relatively healthy, no obvious bacterial infection occurred, and the implant in the mouth was stable. Immediate implantation is beneficial to protect alveolar bone spacing and other tissues, which is conducive to the growth process of attached gingiva.

At 6 and 12 months after repair, the bone resorption in immediate group was significantly less than that in delayed group. Studies have shown that the height of natural gingival depends on the height of natural alveolar. After extraction of the affected tooth, the stress of alveolar ridge changes greatly, and the growth, absorption and reconstruction of alveolar bone are also affected. The natural alveolar bone lost the normal tooth bite force and affected the cortical bone absorption process. When implant is implanted, the dead cells on the implant surface will be necrotic, leading to the occurrence of osseointegration and the absorption of cells 18. At first, with vascular growth, the corresponding periosteal cells recalled the migration of implant surface. Under the combined action of osteoclasts and osteoblasts, bone resorption is affected by many aspects, such as growth factors, and local blood supply 19-20. In the delayed group, the residual alveolar socket after tooth extraction causes partial absorption of the surrounding alveolar during healing, and in the later stage of implantation, a certain amount of bone is removed when preparing the implant socket. Immediately planting on the basis of alveolar socket to prepare planting socket can save the degree of bone grinding and reduce and reduce the absorption of alveolar bone in planting socket. Immediate implant restoration can control occlusal force more reasonably. It can be seen that the immediate implant immediate repair group received reasonable bite force due to physiological stimulation, the surrounding bone mass absorption compared with the delayed group was significantly different.

After 12 months of restoration, the gingival aesthetic index was evaluated. The results showed that the gingival aesthetic effect of immediate group was better than that of delayed group. There was no significant difference in the success rate of implantation between the two groups 12 months after repair. Immediate implantation reduces the absorption of alveolar bone, maintains the height of alveolar ridge, and the bone absorption process is relatively stable, so as to better maintain the height and width of the alveolar. Therefore, the aesthetics is better, and the subjective satisfaction survey of patients is also high.

Amato F et al. 20 showed that immediate implant restoration was superior to delayed implant restoration in improving periodontal tissue health, periodontal pocket depth and aesthetic effect. On the basis of this study, we also observed the differences in the effects of the two planting methods on the buccal keratinized gingival width, alveolar bone absorption and other indicators. The evaluation of the planting effect in this study was more comprehensive, which ensured the credibility of the study.

In conclusion, both delayed restoration and immediate implant restoration can achieve good results in the treatment of maxillary and mandibular molar implant restoration. However, immediate implant has certain advantages in reducing alveolar bone absorption and maintaining gingival aesthetic effect.
